# Molecular Modulators and Receptors of Selective Autophagy: Disease Implication and Identification Strategies

**DOI:** 10.7150/ijbs.83205

**Published:** 2024-01-01

**Authors:** Ming-Yue Wu, Zhe-Wei Li, Jia-Hong Lu

**Affiliations:** State Key Laboratory of Quality Research in Chinese Medicine, Institute of Chinese Medical Sciences, University of Macau, Macau, China.

**Keywords:** selective autophagy, autophagy, screening technology, protein-protein interaction, genome-wide screening

## Abstract

Autophagy is a highly conserved physiological process that maintains cellular homeostasis by recycling cellular contents. Selective autophagy is based on the specificity of cargo recognition and has been implicated in various human diseases, including neurodegenerative diseases and cancer. Selective autophagy receptors and modulators play key roles in this process. Identifying these receptors and modulators and their roles is critical for understanding the machinery and physiological function of selective autophagy and providing therapeutic value for diseases. Using modern researching tools and novel screening technologies, an increasing number of selective autophagy receptors and modulators have been identified. A variety of Strategies and approaches, including protein-protein interactions (PPIs)-based identification and genome-wide screening, have been used to identify selective autophagy receptors and modulators. Understanding the strengths and challenges of these approaches not only promotes the discovery of even more such receptors and modulators but also provides a useful reference for the identification of regulatory proteins or genes involved in other cellular mechanisms. In this review, we summarize the functions, disease association, and identification strategies of selective autophagy receptors and modulators.

## Introduction

Macroautophagy (hereafter autophagy) is a conserved degradation pathway in eukaryotes to help recycle cytoplasmic components to maintain cellular homeostasis 1. The initiation of autophagy is usually subject to the mammalian target of rapamycin (mTOR) inhibition and AMP-activating protein kinase (AMPK) activation, which stimulate the Unc-51 like autophagy activating kinase 1 (ULK1) complex, including autophagy-related protein 13 (ATG13), focal adhesion kinase family interacting protein of 200 kD (FIP200) and autophagy-related protein 101 (ATG101), and facilitate the formation of phagophore fragments 2. ULK1 then transduces autophagic signaling to activate the PI3KC3 complex (BECN1, class III PI 3-kinase (VPS34), PI 3-kinase regulatory subunit 4 (VPS15), autophagy-related protein 14, and nuclear receptor binding factor 2 (NRBF2)) for autophagosome nucleation 3. The production of PI3P and the engagement of WD-repeat protein interacting with phosphoinositides (WIPI) induce the autophagosome expansion. Two ubiquitin-like conjugation modules were required to promote autophagosome formation. First, autophagy-related protein 7 (ATG7) and autophagy-related protein 10 (ATG10) are involved in the conjugation of autophagy-related protein 5 (ATG5), autophagy-related protein 12 (ATG12) and autophagy-related protein 16 (ATG16), which acts as E3 enzyme to stimulate conjugation of phosphatidylethanolamine (PE) to autophagy-related protein 8 (ATG8)-family. ATG7, autophagy-related protein 4 (ATG4) and autophagy-related protein 3 (ATG3) are then responsible for the cleavage of the ATG8-family membranes 4. Lipidated ATG8 ultimately interacts with LC3-interacting region (LIR)-containing proteins, such as receptors and substrates, and proceeds to autophagosome maturation. Following phagophore closure, the autophagosomes fuse with the lysosomes, resulting in the degradation of autophagic substrates by acidic lysosomal hydrolases.

Selective autophagy is the selective form of autophagy, achieved through the specificity of cargo recognition 5. There are various terms to describe diverse selective autophagy according to different autophagic substrates: mitophagy 6, ER-phagy 7, aggrephagy 8, lipophagy 9, xenophagy 10, nucleophagy 11, pexophagy 12, ferritinophagy 13, lysophagy 14 and fluidophagy 15. Studies have revealed that selective autophagy is implicated in various human diseases such as cancers, neurodegenerative diseases, and infectious diseases 16-22. For example, impaired mitophagy has been observed in Alzheimer's disease (AD) models with the downregulation of BCL2 interacting protein 3 (BNIP3), fun14 domain containing 1 (FUNDC1), and optineurin (OPTN) 16, 17. Family with sequence 134, member B (FAM134B), an ER-phagy receptor, plays a tumor-suppressing or tumor-promoting role in different types of cancers, such as colon cancer, esophageal squamous cell carcinoma and pancreatic cancer 18-20. Many selective autophagy stimulators exhibit potential therapeutic effects on diseases 23, 24. Therefore, identification of novel receptors and modulators of selective autophagy is not only necessary for mechanistic studies of the selective autophagy process, but also important for understanding the pathogenesis of many diseases.

In selective autophagy, selective autophagy receptors (SARs) recognize specific cargoes and tether them to the autophagosomes by interacting with the ATG8/LC3 family 25. Typically, an LC3 interacting region (LIR) and a cargo-binding site, such as Ub-binding domains (UBDs), are two necessary components in SARs. FAM134B, NIX, OPTN, TAX1BP1, CCT2, etc., are all well-known selective autophagy receptors with typical or atypical LIRs 26. SARs directly affect the recognition and binding of cargos and autophagosomes. Unlike SARs, selective autophagy modulators usually do not directly recruit the autophagosomes but regulate the process of selective autophagy, such as WIPI2, PTEN-L and Parkin 27, 28. Some of the soluble autophagy modulators tether to the substrates indirectly by recognizing polyubiquitin on the substrates, while parts of autophagy modulators bind to the substrate directly, accompanied with post-translational modifications (PTMs) 4, 29-32. Selective autophagy modulators can also utilize other motifs, for example, ubiquitin-interacting motifs (UIM) bound to Atg8/LC3 proteins and ubiquitin-associated (UBA) domains 33. Taken together, selective autophagy is a comprehensive regulatory process and understanding the modules of selective autophagy is the essential parts for mechanistic exploration of selective autophagy. And due to the structural properties of SARs, new SARs can be found by protein-protein interaction-based screening strategies, while by genome-wide screening both SARs and selective autophagy modulators can be found. Different screening strategies determine whether it is possible to discover these proteins. Our review summarizes screening strategies and technologies used to identify selective autophagy receptors and modulators, and we discuss the strengths and challenges of these strategies and technologies.

## Selective autophagy modulators and receptors in human diseases

**Figure 1:**
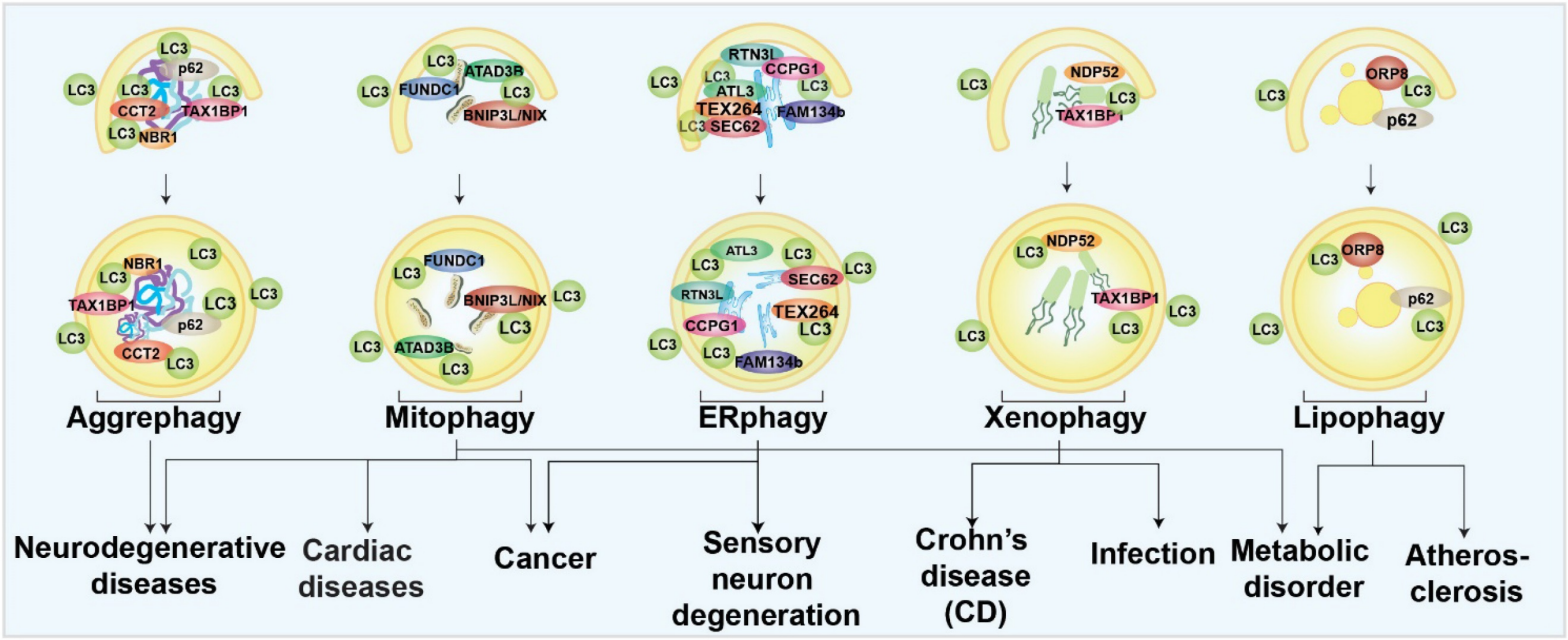
Schematic diagram of selective autophagy modulation in human diseases.

In selective autophagy, certain autophagy receptors target specific autophagic cargoes 34. These receptors bind autophagy cargoes and ATG8s on the inner membrane surface of phagophore and drive the core autophagy process. Autophagy receptors can be ubiquitin-dependent or not. Ubiquitin-dependent selective receptors, such as sequestosome 1 (p62), Neighbor of BRCA1 gene 1 (NBR1), NDP52/CALCOCO2, Tax1 binding protein 1 (TAX1BP1), and OPTN, are the most well-studied 32, 35. These receptors contain oligomerization domains, ATG8-binding LIR domains, and ubiquitin-binding domains that facilitate receptor recognition of substrates 36-39. Among the ubiquitin-independent receptors 29, the glycophagy receptor starch binding domain 1 (STBD1) has a carbohydrate-binding domain for cargo identification. In contrast, the ferritinophagy receptor nuclear receptor coactivator 4 (NCOA4) binds directly to ferritin 38-40. Understanding the roles of selective autophagy in human health and diseases can be inspired by the studies of its receptors and modulators. The different types of selective autophagy involved in different human diseases are briefly summarized in Fig. 1. Several forms of selective autophagy, such as lysophagy, pexophagy, fluidophagy, etc., are not mentioned here, due to the limited evidence to support the disease associations.

### Aggrephagy

Aggrephagy is characterized by specific autophagic degradation of protein aggregates or inclusion bodies within the cell 41. Studies found that targeting certain aggrephagy receptors was a potential treatment for neurodegenerative diseases due to the wide-spread recognition of misfolded protein aggregates as pathogenic factors 42. For example, the lysosomal protein, TECPR1, has been found to facilitate aggrephagy and degrade chemical-induced or overexpressed huntingtin (HTT) protein aggregates neural cells 43. Recently, chaperonin-containing TCP-1 subunit 2 (CCT2) has been identified as novel receptor for solid protein aggrephagy through a LC3-labeled HTT inclusion bodies purification strategy followed by proteomic determination 44. Some potent pharmacological aggrephagy inducers have also been revealed and show neuroprotective capabilities in neurodegenerative diseases, such as PD180970 and XCT 790 45-48.

### Mitophagy

Mitochondria are specific organelles with their own genomes; they govern cellular energy supply 49. Once mitochondria are damaged, mitophagy will be invoked and damaged mitochondria will be specifically eliminated to maintain cellular homeostasis. Mitophagy has been mostly studied especially in neurodegenerative diseases 50. Mice lacking Parkin- and Pink-dependent mitophagy are found to have an accumulation of mutant mtDNAs, which may be a pathogenic factor for PD 51. Mitophagy is also related to AD. In a recent work, our group used machine learning to identify two novel small molecules as mitophagy enhancers that have demonstrated the capacity to improve AD-associated pathologies 52. Mitophagy has also been discussed extensively in cancer therapy due to the accumulation of reactive oxygen species (ROS) and mtDNAs caused by mitochondrial dysfunction. The elevated toxins, such as ROS, mtDNA, ATP, and N-formyl peptides from damaged mitochondria have been shown to increase genomic instability and accelerate the development of cancer 53. Some mitophagy-related genes, such as *BNIP3* and *PARKIN*, act as tumor suppressors 53, 54. Mitophagy inhibition can be effective in cancer treatment if mitophagy is considered as an adaptive process. Mitophagy inhibitors, such as liensinine and MDIVI-1, have been found to enhance the sensitivity of tumor cells to chemotherapy 55, 56. In the aging heart, mitophagy has been regarded as the center of rejuvenation 57. Mitophagy defects exacerbated cardiac injury and reduced survival following myocardial infarction in one *in vivo* study 58. Meanwhile, mitophagy-associated proteins, such as FUNDC1 and RAB9, have been proved to be protective in heart ischemia models 59, 60. Mitophagy can also trigger muscle-adipose crosstalk to alleviate dietary obesity 61. Mitochondria are tightly coupled with cellular metabolism, and mitophagy has emerged as a player in metabolic diseases. Studies have found that mitophagy in adipose tissue increases cellular lipid metabolic processing capacity. The diabetes susceptibility gene *CLEC16A* is involved in mitophagy regulation in β cells 62-64. In summary, regulation of mitophagy might be a promising strategy for many diseases.

### Endoplasmic reticulum (ER)-phagy

Endoplasmic reticulum (ER)-phagy is an autophagic process to degrade specific parts of the ER for ER quality control and function maintenance 65. By analyzing ER-phagy-related proteins, researchers determined that ER-phagy is closely associated with a series of neurological disorders and cancers. In mammalian cells, FAM134B, Reticulon-3 (RTN3L), SEC62, cell cycle progression 1 (CCPG1), atlastin GTPases 1 (ATL1), and testes expressed gene 264 (TEX264) are well-known ER-phagy receptors 65. Most of the ER-phagy-related genes were found to function in the neurological system. Mutations of ATL1 have been found to be a cause of hereditary sensory neuropathy type 1 66. FAM134B is necessary for long-term survival of nociceptive and autonomic ganglion neurons 67. RTN3 is involved in the etiology of AD 68. In summary, ER-phagy appears to be closely linked to neurological function, especially regulation of sensory neurons. Dexmedetomidine, a sedative, has been proved to induce ER-phagy thereby alleviating ER stress in a spinal cord neuropathic pain model 24. Cancers are also linked to ER-phagy. Studies have found that Sec62 is a potential oncogene in non-small cell lung cancer 69, and is proposed as a prognostic marker in advanced head and neck squamous cell carcinoma 70. Moreover, novel FAM134B (JK1) mutations have been identified in oesophageal squamous cell carcinoma 19. Although many ER-phagy regulators have been found to be involved in several diseases, exactly how ER-phagy is related to these diseases, in terms of mechanism, is not understood.

### Xenophagy

Xenophagy, which targets bacterial pathogens for lysosomal degradation, has been mostly linked to infectious diseases as a host defensive mechanism 10, 71. Xenophagy is closely related with Crohn's disease (CD), a kind of inflammatory bowel disease type. Numerous proteins, including ATG16L1, immunity-related GTPase M (IRGM), and nucleotide-binding oligomerization domain 2 (NOD2), have been linked to the development of CD 72-75. Patients with severe Covid-19 have also shown signs of virus-induced xenophagy 76. It has been demonstrated that increased xenophagy helps to protect the host against infection. For example, hypoxia-inducible factor (HIF-1) is a mediator to activate xenophagy and limit salmonella infection and spread 21. Resveratrol also activates xenophagy and promotes intracellular bacteria clearance in intestinal epithelial cells and macrophages 77.

### Lipophagy

The selective autophagy that targets lipid droplets is called lipophagy 9. Since metabolic disorders frequently involve lipid droplet build-up, controlling droplet degradation has been proposed to alleviate lipid stresses 78-81. Lipophagy is thought to regulate the breakdown of lipid droplets in the liver, and much research have examined the functions of lipophagy in regulating the progression of non-alcoholic fatty liver disease (NAFLD) 78, 82, 83. Elevating lipophagy alleviates NAFLD. For example, iridoids and phillygenin have been identified as lipophagy enhancers and have been found to ameliorate NAFLD and hepatic steatosis 82, 83. However, lack of hepatic autophagy in mice was resistant to physiological steatosis due to activation of nuclear factor erythroid 2-related factor 2 (NRF2) and maintenance of nuclear receptor corepressor 1 (NCoR1) 84-86, indicating that compensatory regulations may be upregulated in the organistic system, especially inhibition of fasting-induced de novo lipogenesis in the liver. Lipophagy is also critical to maintaining the lipid homeostasis of vascular endothelial cells, vascular smooth muscle cells, and macrophages. As lipid accumulation in these cells is closely related to atherosclerosis, induction of lipophagy may have therapeutic value for atherosclerosis 80. Multiple sclerosis and diabetic nephropathy are also linked to lipophagy. Thus, targeting lipophagy shows extensive potential to regulate wide range of serious diseases 79, 87.

## Screening for selective autophagy receptors and modulators

As targeting selective autophagy is promising as a strategy to treat diseases, it is important to understand disease pathogenesis in terms of autophagy receptors and modulators. Screening autophagy receptors and modulators using various modern technologies is an efficient methodology in this regard. We will briefly summarize and discuss emerging screening methods in sections below.

In selective autophagy receptor and modulator identification, the first step is usually establishment of robust reporters to monitor selective autophagy. For example, GFP-mcherry-LC3, mito-keima, and mcherry-eGFP-RAMP4 are all reporter systems for autophagy and selective autophagy determination, which have been successfully used for modulator screening 88-90. Other fluorescent reporters which have not applied in autophagy regulator screening are also expected to be used, such as Ribo-Keima reporter 91. The second step is to establish specific selective autophagy induction conditions 92, 93. The third, last step is to use target-based, or phenotype-based approaches 38, 94, 95. Selective autophagy receptors and modulators can also be identified by isolating subcellular organelles or components from cells with different levels of autophagy, such as lipid droplets (lipophagy) 94 and inclusion bodies (aggrephagy) 44.

**Figure 2:**
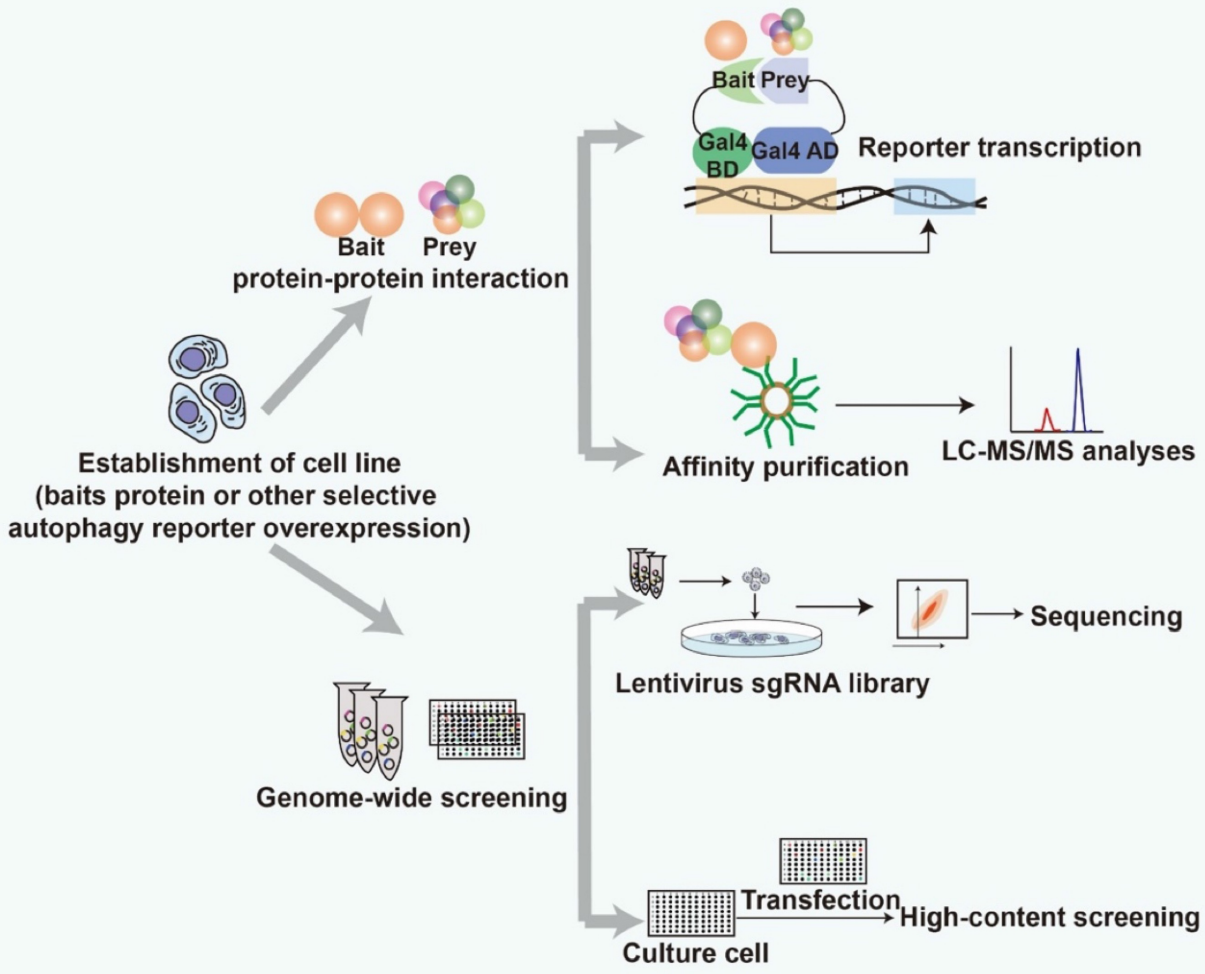
Schematic representation of strategy for screening selective autophagy receptors and modulators.

Strategies based on protein-protein interactions (PPIs) and genome-wide screening have been used to find specific selective autophagy receptors and modulators (Fig. 2). Both strategies require robust detection methods to screen for the receptors and modulators. The identified proteins can be defined as selective autophagy receptors and modulators after further experimental validation, such as co-immunoprecipitation, fluorescence co-localization and functional characterization. Transmission election microscopy (TEM) is a gold standard to assess the status of autophagy. Recently, it has been successfully applied to analyze peripheral autophagy markers associated with PD 96. TEM is recommended to be used to evaluate the efficacy of the screened receptors and modulators and investigate their functional implications for autophagy.

### Approaches based on protein-protein interactions (PPIs)

Autophagic related proteins form functional complexes to achieve the fine regulation of the autophagy process. For example, ULK1-FIP200-ATG13, ATG14L-BECN1-VPS34-VPS15, and ATG5-ATG12-ATG16 are three protein complexes involved in autophagy initiation, autophagosome elongation, and maturation, respectively. In selective autophagy, receptor-cargo recognition is a critical step to obtain specificity 25. Based on the recognition modules of selective autophagy receptors, an efficient strategy for objectively identifying novel selective autophagy receptors, is bait-based identification of interacting proteins. Atg8/LC3 family proteins are widely utilized as bait 43, 44, 93, 97-104. The bait-interacting proteins or proteins with LIR motifs are candidate receptors for further validation 105. In recent years, strategies based on PPIs are widely used to identify selective autophagy regulators 94, 101, 105-107. Three strategies will be described below: computational LIR prediction, yeast two-hybrid (Y2H) screening, and mass spectrometry-based proteomic analysis.

#### Computational LIR prediction

Atg8-family proteins play a central role in mediating selective autophagy 105. Some of the well-known selective autophagy receptors, such as p62, NBR1 and BNIP3, achieve autophagosome targeting by binding with LC3 at its interacting region (LIR) 32. Jacomin, et al., have developed a free database to predict new LIR-containing proteins (LIRCPs) using a computational approach 105, 108. Many regulators have been successfully identified using this method. In 2019, Yifan Zhang, et al., revealed that *Listeria monocytogenes* can induce mitophagy independent of known mitophagy receptors (Nix, BNIP3 and FUNDC1) 109. Applying an iLIR web server, they successfully screened and identified a novel mitophagy regulator, NLR family member X1 (NLRX1), which contains an LIR motif in the pattern-recognition receptors (PPRs). Computational prediction is an easy approach to screening for receptors that contain the LIR motif. However, it's not able to predict the noncanonical LIR motif-containing receptor, such as CALCOCO2/NDP52 110 and additional procedures required to be done to get convincing results.

LIR is a short linear motif (SLiM) present in many proteins, including those that do not interact with LC3 111. To eliminate some spurious LC3-binding proteins, intrinsically disordered regions are required to be identified. The Disprot-Database of Protein Disorder (http://www.disprot.org/pondr-fit.php) is a free website for confirming disordered regions. It's better to exclude disordered regions on Disprot before prediction. In the cases without typical LIR, and updated prediction based on the artificial intelligence (AI) system, named AlphaFold2-multimer, can be used 112. This newly established AI system has successfully predicted some atypical LIR motifs, such as ILVV in NDP52, MLVV in TAX1BP1, and YDFM in ATG40. Post-translational modification (PTM) is an important manner to regulate LIR-binding efficiency. Phosphorylating of S177 adjacent to the LIR of OPTN and S332 in the LIR motif of p62 may regulate the LC3 binding efficiency 113, 114. In addition, acetylation of Lys49 on the LIR has been found to disrupt the LC3 binding 115. Therefore, the prediction of the PTM domain in LIR could be an easy way to find out the potential PTM-related regulatory pathway. There are several online databases and related tools for PTM prediction. For example, DAPPLE (http://saphire.usask.ca/saphire/dapple2) focuses on phosphorylation modification, dbPTM (http://dbPTM.mbc.nctu.edu.tw/) can predict the modifications of phosphorylation, glycosylation and sulfation, and PTM-ssMP (http://bioinformatics.ustc.edu.cn/PTM-ssMP/index/) that can predict different types of modifications. All these prediction servers can be useful tools for screening and greatly assist in the identification of PTM-associated regulators in selective autophagy.

#### Yeast two-hybrid (Y2H) screening

The yeast two-hybrid (Y2H) system is a well-established genetics-based system to screen for bait-binding proteins from a large-scale cDNA library 116. In selective autophagy study, bait proteins are fused with the DNA-binding domain (BD) of Gal4. The Gal4 activation domain is fused to prey. Reporter gene expression indicates successful interaction of bait proteins and prey proteins. Atg8 family proteins mostly used bait 117-120. A successful example was the identification of ER-phagy receptor, FAM134^98^. In this case, LC3B and GATE16/GABARAPL2 are bait. After screening, FAM134 family members were identified 98. FKBP prolyl isomerase 8 (FKBP8) was also identified as a mitophagy regulator in a human thymus cDNA library screening using LC3B as bait 100. Proteins other than those in the LC3 family were also designed as bait for screening selective autophagy regulators 121-123. ZZ and LB domains of p62 were used as bait in yeast two-hybrid screening. The prefoldin-like chaperone ubiquitously expressed transcript protein (UXT) was identified as an aggrephagy adaptor that binds to the LB domain of p62 123. In a xenophagy study, NLR family pyrin domain containing 4 (NLRP4) was used as bait, and ARHGDIA was finally identified as a regulator protein during Group A* Streptococcus* (GAS) infection 124. Compared to the *in vitro* approaches based on bacterial expression, Y2H is a more advanced *in vivo* technique using yeast as a host cell 125. However, not all modification-driving interactions can be observed by using this method. The construction of cDNA libraries is challenging, and the screening process is time-consuming and labor-intensive.

#### Mass spectrometry (MS)-based proteomic analysis

MS-based proteomic analysis is a powerful approach that has been widely used in the identification of receptors and modulators that bind with bait proteins. Proteomics analysis unbiasedly provides plenty of protein information by its large-scale protein screening capacity and flexible application with different labeling methods 25. Another advantage is that proteomics can read the protein composition comprehensively without utilizing conventional fluorescence labelling-based autophagy assays 126.

##### Affinity purification coupled with MS-proteomics

Affinity purification is widely used for selective autophagy study. In selective autophagy study, Atg8 families (LC3s), selective autophagy receptors, or selective autophagy substrates are usually chosen as bait proteins for affinity purification 93, 106, 127. Positive hits are analyzed and subjected to secondary screening to sort out those potentially involved in selective autophagy.

In most cases, ATG8 families are widely used as bait proteins for screening selective autophagy regulators. By expressing YFP-FLAG-His6 (YFH)-tagged ATG8 in *Schizosaccharomyces pombe*, two research groups performed affinity-purification coupled with mass spectrometry (AP-MS) analysis and then identified an ER-phagy receptor, Mug185 93, 128. Using ATG8 as a bait in *Saccharomyces cerevisiae*, autophagy-related protein 39 (ATG39) and autophagy-related protein 40 (ATG40) were identified as receptors for nucleophagy and ER-phagy, respectively, after mass spectrometry analysis of ATG8 immunoprecipitates 106. In plants, researchers have used tunicamycin to trigger stress-induced-ER-phagy and immunoprecipitation-MS analysis to obtain the GFP-ATG8A binding proteins. In this work, cytosolic protein C53 was identified as an ER-phagy regulator 99. To identify PolyQ-huntingtin (HTT) inclusion bodies (IBs)-associated aggrephagy regulator, IBs with high (H) and low (L) LC3 recruitment were isolated by a fluorescence-activated particle sorting (FAPS) system, which can enhance the accuracy of screening. Then by mass spectrometry, CCT2 was identified as a novel aggrephagy regulator that interacted with ATG8s through a VLIR motif 44.

Besides the Atg8 family, autophagy receptors and substrates have also been applied for IP-MS analysis. To identify novel regulators associated with p62 or autophagy-linked FYVE protein (ALFY), one study compared the immunoprecipitated proteins from ALFY^-/-^ vs WT cells, or from EGFP-p62 over-expressing cells vs EGFP over-expressing cells. The study finally identified two proteins, nipsnap homolog 1 (NIPSNAP1) and nipsnap homolog 2 (NIPSNAP2, that selectively interacted with ALFY and p62 and were further confirmed as mitophagy regulators 129. Another study performed co-immunoprecipitation (Co-IP) to obtain the zinc finger FYVE-type containing 1 (ZFYVE1) or WIPI1-associated proteins after inducing mitophagy. In this study, breast carcinoma amplified sequence 3 (BCAS3) and chromosome 16 open reading frame 70 (C16orf70) were identified as selective and non-selective autophagy regulators, respectively 127.

More specifically, mutation in the binding domain of LC3B has been established for identification of interacting proteins. One study used K51A mutant LC3B to screen for novel LC3B-interacting autophagy regulators 101. Researchers identified TEX264 as having a high binding score by analyzing the IP-MS results using LC3B WT and the LC3B K51A mutant proteins as bait. Subsequently, TEX264 was determined as being an ER-phagy receptor by its cellular localization and by other confirmation experiments.

The Immunoprecipitation-Mass Spectrometry (IP-MS) may also identify unexpected receptors and modulators. For example, to figure out the new function of mitochondrial fission protein 1 (Fis1), flag-tagged Fis1-expressing Hela cells were used to identify proteins that it interacted with 90. Syntaxin 17 (STX17), a soluble N-ethylmaleimide-sensitive factor attachment protein receptors (SNARE) protein, was identified as a novel regulator interacting with Fis1. Functional study showed that Fis1 restrained the localization of STX17 onto mitochondria and ER-mitochondria contact sites to prevent STX17-mediated mitophagy. Ribophagy receptor, nuclear fragile X mental retardation-interacting protein 1 (NUPFIP1), was identified after analyzing the mTORC1-regulated lysosomal proteome 130. Transmembrane protein 192 (TMEM192) is a lysosomal/endosomal protein, which has been used as a marker to target and pull-down lysosomal proteins in this study. Researchers utilized 3-HA-taged TMEM192 or 2xFLAG-tagged to perform IP after triggering autophagy by starving or treating with Torin 1, then, from the proteomic results, defined the mTORC1 dependent-lysosomal proteins. At last, they confirmed nuclear fragile X mental retardation-interacting protein 1 (NUFIP1) as a ribophagy receptor that binds to LC3B to regulate ribosomal degradation.

##### MS-proteomic analysis with advanced labeling methods

Despite being a powerful tool in biological study, conventional proteomic analysis is not highly quantitative 131. Additionally, weak, or temporary binding partners are difficult to detect based on original MS conditions. To solve these problems, stable isotopes are incorporated into proteins or peptides to achieve accurate and sensitive quantification. By labeling proteins or peptides in a specific group, the stable isotope labeling by amino acids in cell culture (SILAC) approach allows researchers to perform highly quantitative analysis of proteomic changes in two or more different groups. Moreover, the novel proximity labeling-MS method can label “prey” proteins near the bait and detect transient and weak binding partners 132.

SILAC is a commonly used approach for quantitative analysis 133. Metabolic labeling allows analyzation of cell mixtures from two different groups as a single sample, which greatly reduces the variation of quantitative results due to subsequent experimental procedures and can be used to detect low-level protein alteration. This method can provide condition-dependent quantitative information. A classic example was the identification of NCOA4 as the cargo receptor mediating ferritinophagy 38. In this study, PANC-1 and PA-TU-8988T cell lines and MCF7 cell line with different autophagy levels were studied. Autophagosomes were purified by density gradient separation and subjected to MS analysis. NCOA4 was identified as ferritinophagy receptor based on candidate overlap. Interestingly, later analysis of MS data from this research identified CALCOCO1 as an ER-phagy receptor 134. To identify autophagy dependent ATG9A-interacted proteins, stable isotope labeling by amino acids (SILAC) was applied to nutrient-rich and amino acid-starved ATG9A-overexpressing HEK293 cells. MS-based proteomic analysis of immuno-isolated ATG9A-positive membrane proteins revealed that BAR domain-containing proteins were associated with ATG9A vesicles 135.

Proximity labeling- MS is an effective approach to identify regulatory proteins with weak transient interactions that can be used to bait proteins. Diverse enzymes, such as biotin ligases, PTM ligases, and peroxidases, are utilized for labeling neighboring proteins 136, 137. Peroxidases label proteins by converting biotin phenol to a biotin-phenoxyl radical in presence of H_2_O_2_ 25. MS coupled with proximity-dependent biotin identification (BioID) has been used to study known and novel protein-protein high-confidence proximity interactions in the autophagy pathway. For example, a tandem MS was performed among BioID bait proteins within the autophagy pathway before streptavidin purification. Subsequently, spermine binding protein-like (SBPL) family proteins were identified as Atg8-family interacting proteins in selective autophagy 138. In a study, co-expressing HA-tagged hATG8s and BioID-tectonin beta-propeller repeat containing 1 (TECPR1) has been used to analyze biotinylation of hATG8s in cell lysates. Finally, researchers found that LC3C was strongly biotinylated and TECPR1 selectively interacted with LC3C to facilitate autophagosome-lysosome fusion to promote aggrephagy 43. Proximity labeling-mass spectrometry provides an abundance of protein interaction information. In Wei, L. et al.'s study, APEX2 was used as a bait to identify potential cargos of SQSTM1-like receptors (SLR) in autophagosomes. In this study, a panel of Hela cell lines overexpressing NBR1, NDP52, OPTN, p62, TAX1BP1, and toll interacting protein (TOLLIP) that fused with apurinic endodeoxyribonuclease 2 (APEX2) was established. These APEX2 overexpressing cells were treated with BafA1 prior to H_2_O_2_. TMEM106B is an endosome/lysosome marker, and the authors first established a TMEM106B-APEX2 over-expressing cell line to optimize the vesicle content profiling and then used APEX2-LC3B-expressing cells as an autophagy control. Above 200 proteins were filtered and identified as proximity partners of SLRs and LC3B 139.

SILAC and proximity labeling has been utilized together to distinguish enrichment proteins in specific conditions. Using SILAC-labeled AP2-hATG8s-overexpressing cells in the absence and presence of bafilomycin A1 (BafA1) prior to addition of H_2_O_2_ can enrich the biotinylated proteins in autophagosomes. The mitochondrial protein metaxin 1 (MTX1) has been identified as an autophagosome cargo in mitophagy 97. MS is not only widely used in identifying protein-protein interactions, but also applicable for cell proteomics, organelle proteomics and post-translational modifications detection to achieve unbiased screening of selective autophagy regulators. S. Robichaud et al. isolated lipid droplets (LDs) from macrophage foam cells under basal conditions or upon chloroquine treatment and then performed MS. By comparing the proteomes of chloroquine-treated LDs and untreated LDs, they identified several lipophagy regulators, most of which contain LIR motifs 94. To isolate mono-Ub modified proteins in the identification of ubiquitylation-modified regulators upon dengue infection, J. S. Zhang et al. utilized purified HA-Ub-L73P∗. Among 40 candidates, a lipid droplet protein, ancient ubiquitous protein 1 (AUP1), was identified as a lipophagy regulator 140.

PPIs provide important information for understanding selective autophagy machinery. The general approach is to design autophagy or selective autophagy-related protein bait, and then to screen and identify bait-associated proteins in different conditions. MS-based proteomic analysis is the most widely used approach for protein identification. Using tag-associated “bait” protein overexpressing cells can enrich “prey” proteins for further MS-based protein identification. The approach can also be combined with other advanced labeling techniques for more informative analysis. However, how to obtain high-confident candidates from large amounts of data is a challenge. In addition, MS is a necessary technique for PPIs, but not all proteins can be covered by MS, which can be a major limitation in application.

### Genome-wide selective autophagy screening

Another effective and objective method for screening selective autophagy receptors and modulators is the functional genomics screening approach, which is an unbiased strategy to identify genes or genetic elements related to phenotype of interest 141, 142. High-throughput loss-of-function screening and identification of selective autophagy receptors and modulators based on cellular phenotypes has been well established in recent years. Genome-wide RNAi and yeast library screening are two methods dependent on a large library data pool. In recent years, CRISPR-Cas9-based screening has rapidly developed, and this method has been widely used in selective autophagy modulator screening 22, 103, 143, 144.

#### RNAi-based screening

RNAi-based screening is usually performed in an arrayed or pooled format. By combining high-content screening or high through-put screening platforms, it can efficiently identify regulator proteins 102, 145-147. Mandell et al. transfected mRFP-GFP-LC3B Hela cells with SMARTpool siRNAs to screen functional regulators of autophagy according to the RFP and GFP puncta. TRIM5α was identified as a selective autophagy receptor of HIV-1 capsid in this study 146. TRIM has also been identified as a selective autophagy receptor for inflammatory proteins in another similar study 102. In this study, TRIM family was silenced using SMARTpool siRNA in THP-1 cells. Image-based high-content analysis of LC3 puncta revealed TRIM family proteins were involved in IFN-γ induced-autophagy. They found that tripartite motif-containing (TRIM) family proteins can selectively target key components for autophagic degradation in inflammasome and type I interferon responses 102. A directed siRNA screening of 67 lipid kinase and phosphatase genes was performed to identify the lipid kinases and phosphatases regulating xenophagy. By evaluating the changes of host bacterial defense and further experiments, SAC1 was identified as a xenophagy regulator 148. However, RNAi-based screening is limited by the relative high cost of siRNA library, and the excessive amounts of time and labor require.

#### CRISPR-Cas9-based screening

As CRISPR-Cas9-based genome editing methods have developed, the CRISPR-Cas 9 system has been quickly adapted for functional genomic screening. Compared with RNAi pool screening, the CRISPR-Cas9 technique is more efficient for genome screening, and it can deliver quantitative results 149. Liang JR et al. have established an ER-phagy tandem reporter (EATR) system, and performed genome-wide flow cytometry-based CRISPRi screening for ER-phagy regulatory genes 144. The screening successfully identified mitochondrial metabolism and ER-resident UFMylation related genes involved in ER-phagy 89. This approach can analyze the ER-phagy-regulatory pathway and modifications systemically and without bias.

By screening IFN-β-EGFP reporter cell lines with CRISPRi, coiled-coil domain-containing protein 50 (CCDC50) was identified as a negative regulator for IFN-β expression 22. Further evidence proved that CCDC50 was a selective autophagy receptor participating in retinoic acid-inducible gene 1 (RIG-I)/MDA5 degradation during viral infection. To identify xenophagy regulatory factors, Yue Xu et al. performed CRISPRi screening of FIP200^-/-^ GFP-LC3 Hela cells expressing LAMP1-mCherry 103. FIP200 is a canonical autophagic protein but is dispensable in bacteria-induced autophagy. FIP200 deficiency cell type is a good sorting cell type to avoid basal autophagy flux interference, but not bother xenophagy formation. The xenophagy-positive cells were sorted after BFP-labeled bacterial infection, and V-ATPase was identified as a xenophagy regulator.

#### Genome-wide yeast library screening

The genome-wide yeast library has also been used for screening selective autophagy regulators. To investigate ER-phagy regulators, yeasts were transformed with Sec61-mCherry or Rtn1-GFP plasmids and positive clones were identified based on imaging analysis when the fusion of mCherry and vacuoles was impaired after rapamycin stimulation. GFP-Atg8 was used as a control to assess whether these mutants lead to defects in bulk autophagy. This study identified Vps13 as one of the regulators of ER-phagy. Further mechanistic studies showed that Vps13 mutant cells exhibited a 70% reduction in the packaging of the ER into autophagosomes but didn't alter the number of autophagosomes 104. In another study, authors used ER-shaping mutant yeasts to identify whether ER-shaping proteins play a role in ER-phagy. ER-phagy receptor Sec61-GFP was utilized. And after treating cells with rapamycin (mutant clones failed to increase GFP puncta), Lnp1 was finally identified based on imaging analysis. Further evidence proved that Lnp1 was required to stabilize the actin-dependent ER remodeling in ER-phagy 150. Using ATG gene knock-out yeast library, autophagy-related protein 24 (ATG24)/SNX4 was identified to be required for selective autophagic degradation of proteasomes 151.

Other forward genetic screening studies have been used for selective autophagy regulator screening and identification 152-154. Screening of a Drosophila eye lethal mutant library revealed that Tre-2/Bub2/Cdc16 (TBC) protein dTBC1D22 modulated Rab40 to regulate lipophagy 81.

The RNAi technique and yeast mutant library which are usually handled as pre-setting array formats limit researchers to choosing detection methods, such as high-content imaging analysis with pooled population 102, 104, 150. In comparison with these two approaches, the CRISPR-Cas9-based technique is more flexible in that cell phenotype detection methods can be chosen without format limitation. However, dependence on flow cytometry-based fluorescence detection restricts the selection of models 22, 103, 144.

Genome-wide screening is undoubtedly a powerful tool to screen for selective autophagy-related genes. It can screen out genes that are associated with changes in selective autophagy. However, the candidate genes may not encode receptors and modulators in selective autophagy, and further biological confirmation is required for refinement. PPI may be a better way to identify specific selective autophagy receptors or modulators, but low abundance proteins may be missed due to the limitation of the technique. Combination of multiple screening strategies may enhance the target identification efficiency and facilitate off-targets deconvolution process.

## Summary and future perspectives

Identification of selective autophagy receptors and modulators is critical for understanding the selective autophagy machinery and regulatory network. Our review summarizes current screening strategies and technologies applied in the discovery of selective autophagy receptors and modulators and discusses the advantages and disadvantages of these technologies. It is a resource for the selection of strategy and technology to be used in future studies. However, most of the screening models used to identify interaction partners involve over-expression of selective autophagy markers. We foresee that, in the future, label-free technologies, like cellular thermal shift assay (CESTA), will be developed 157, 158.

An increasing number of tools to monitor selective autophagy have been developed. Before applying these new tools in screening, it is important to confirm the reliability and sensitivity of the tools. For example, ER-phagy probe GFP-mCherry-RAMP4 indicates a higher level of ER-phagy when there are more red-only puncta. However, flow cytometry results indicate that the ratio of red to green puncta must be used, rather than the number of red puncta. Fluorescent images provide more dimensional information regarding the distribution pattern of interested probes, but efficient quantification from a powerful analytic system is required for screening. Fortunately, the latest high-content imaging systems are equipped with powerful image analysis tools that can perform quantification analysis. Recently, artificial intelligence (AI) technology has also been successfully applied in image analysis, allowing more efficient and accurate image quantification 159, 160. Although an increasing number of selective autophagy small molecule compounds have been identified, most of them fail to precisely regulate a specific form of selective autophagy without affecting other forms of autophagy. For example, carbonyl cyanide m-chlorophenyl-hydrazone (CCCP), the most commonly used mitophagy inducer, induces both mitophagy and bulk autophagy 161. The limitation in selectivity results in misleading data that confuses the screening analysis. In future studies, more effort focused on the identification of highly selective and druggable receptors and modulators, including kinases and GPCRs, for selective autophagy regulation will enable us to achieve the goal of efficient modulation of selective autophagy under physiological and pathological conditions.

## Figures and Tables

**Table 1:** Comparison of different screening approaches.

Forms of selective autophagy	Methods description		Hits	Verified receptors and modulators	Reference
	Bait/Target	Screening method			
ER-phagy	ATG8	MS-proteomic	>400	Epr1	93
	Sec61, Rtn1 and ATG8	Genetic Screening	21	Vps13	104
	Sec61	Genetic Screening	/	Lnp1	150
	LC3B and GABARAPL2	Y2H Screening	/	FAM134	98
	ATG8	MS-proteomic	/	C53	99
	RAMP4 and LC3B	CRISPRi Screening	200	RPN1, etc.	89
	LC3B	MS-proteomic	87	TEX264	101
	ALFY and p62	MS-proteomic	>11	NIPSNAP1 and NIPSNAP2	129
Mitophagy	Fis1	MS-proteomic	/	STX17 and Fis1	90
	/	LIR prediction	/	NLRX1	109
	ATG8	Y2H Screening	/	FKBP8	100
	PRKN, ZFYVE1 and WIPI1	MS-proteomic	35	BCAS3 and C16orf70	127
	Om45	Genetic Screening	/	YIL146C, ECM37, and ATG32	155
	Clec16a	MS-proteomic	800	Nrdp1	64
	ATG8s	MS-proteomic	1147	MTX1, LC3C	97
	OPTN	CRISPRi Screening	273	VDAC2, TOMM20, etc.	156
	PARK2	CRISPRi Screening	>50	PARK2, PINK1, aTG14, etc.	156
Aggrephagy	NBR1, NDP52, OPTN, p62, TAX1BP1, and TOLLIP	MS-proteomic	279	autophagy substrates	139
	p62	Y2H Screen	12	UXT	123
	LC3	MS-proteomic	>30	CCT2	44
Xenophagy	LAMP1 and LC3	Genetic Screening	1	SopF, etc.	103
Lipophagy	/	MS-proteomic	91	More than 10	94
	/	MS-proteomic	40	AUP1	140
Ribophagy	mTORC1	MS-proteomic	343	NUFIP1	130
IFN-γ induced autophagy	LC3	RNAi Screening	24	24 different TRIMs	102
Selective autophagy degradation for RIG-I/MD54	IFN-β	CRISPRi Screening	1000	CCDC50	22
